# Multiomics studies with co-transformation reveal microRNAs via miRNA-TF-mRNA network participating in wood formation in *Hevea brasiliensis*

**DOI:** 10.3389/fpls.2023.1068796

**Published:** 2023-08-14

**Authors:** Jinhui Chen, Mingming Liu, Xiangxu Meng, Yuanyuan Zhang, Yue Wang, Nanbo Jiao, Jianmiao Chen

**Affiliations:** ^1^ Sanya Nanfan Research Institute of Hainan University, Hainan Yazhou Bay Seed Laboratory/Key Laboratory of Genetics and Germplasm Innovation of Tropical Special Forest Trees and Ornamental Plants, Ministry of Education, School of Forestry, Hainan University, Sanya, China; ^2^ Engineering Research Center of Rare and Precious Tree Species in Hainan Province, School of Forestry, Hainan University, Haikou, China; ^3^ School of Tropical Crops, Hainan University, Haikou, China; ^4^ Rubber Research Institute, Chinese Academy of Tropical Agricultural Sciences, Haikou, Hainan, China; ^5^ State Centre for Rubber Breeding, Haikou, Hainan, China

**Keywords:** reaction wood, phenylpropanoid biosynthesis pathway, lignin biosynthesis, *Hevea brasiliensis*, miRNA

## Abstract

**Introduction:**

MicroRNAs (miRNAs) are small endogenous non-coding RNAs that play an important role in wood formation in plants. However, the significance of the link between miRNAs and their target transcripts in wood formation remains unclear in rubber tree (*Hevea brasiliensis*).

**Methods:**

In this study, we induced the formation of reaction wood by artificially bending rubber trees for 300 days and performed small RNA sequencing and transcriptome deep sequencing (RNA-seq) to describe the complement of miRNAs and their targets contributing to this process.

**Results and discussion:**

We identified 5, 11, and 2 differentially abundant miRNAs in normal wood (NW) compared to tension wood (TW), in NW relative to opposite wood (OW), and between TW and OW, respectively. We also identified 12 novel miRNAs and 39 potential miRNA-mRNA pairs with different accumulation patterns in NW, TW, and OW. We noticed that many miRNAs targeted transcription factor genes, which were enriched in KEGG pathways associated with phenylpropanoid biosynthesis, phenylalanine metabolism, and pyruvate metabolism. Thus, miRNA-TF-mRNA network involved in wood formation via tension wood model were constructed. We validated the differential accumulation of miRNAs and their targets by RT-qPCR analysis and overexpressed miRNA in *Nicotiana benthamiana* with its potential target gene. These results will provide a reference for a deep exploration of growth and development in rubber tree.

## Introduction

Rubber tree (*Hevea brasiliensis*) is the main source of natural rubber (NR). Once latex production is no longer economically viable, rubber trees are also used as timber. The wood from rubber trees has in fact become the main export commodity in southeast Asia (Nair, 2010). Its delicate color and outstanding physical performance make it an excellent option for flooring and home furnishings. Due to its high potential commercial value, increasing timber yield and quality has become a key point of biotechnology in the rubber tree industry (Priyadarshan, 2017). However, two major limitations hinder a more widespread use of rubber wood: (i) The extent of unlignified or partially lignified tension wood fiber, which is not easily digested by enzymes, is high, and the proportion of normal fibers is low (Mellerowicz and Gorshkova, 2012; Gritsch et al., 2015); and (ii) it has high sensitivity to biodegradation owing to low levels of phenolic compounds with biocidal activity (Pramod et al., 2019; Thaochan et al., 2020). The biosynthesis of lignin and polyphenolic derivatives in living trees, particularly in rapidly growing woody plants such as rubber trees, contributes to wood quality and durability (Kuyyogsuy et al., 2018; Pramod et al., 2019; Thaochan et al., 2020).

Lignin is an abundant biopolymer that is essential for plant cell wall integrity and stem strength (Shen et al., 2012). The biosynthesis of lignin monomers begins with phenylalanine deamination, leading to the production of three monolignin alcohols: coniferyl, sinapyl, and *p*-coumaryl alcohols (Eudes et al., 2012). Several genes that participate in lignin biosynthesis and modulate lignin levels have been identified in dicots (Sibout et al., 2005; Weng et al., 2010). For instance, knocking down *4-coumarate:CoA ligase* (*4CL*) expression in hybrid poplar (*Populus tremula* × *P. alba*) resulted in a sharp decrease in lignin content and markedly altered wood chemical composition and wood metabolism (Voelker et al., 2010). The *PtrWND* (*wood-associated NAC domain*) genes were shown to induce the expression of wood biosynthetic genes, including associated structural genes and transcription factors, resulting in ectopic deposition of lignin in poplars (Zhong et al., 2010). Despite the above progress in our understanding of lignin biosynthesis, much remains to be investigated in terms of transcriptional and post-transcriptional regulation. Recently, regulation of wood formation via non-coding RNAs (ncRNAs) and microRNAs (miRNAs) has received increasing attention (Lu et al., 2013).

miRNAs are post-transcriptional modulators of gene function by promoting the cleavage of their complementary target messenger RNAs (mRNAs) and/or imposing translation repression (Bartel, 2004; Zhang et al., 2019). Several studies have illustrated the vital roles played by miRNAs in wood formation (Li et al., 2020; Yu et al., 2020). For instance, transcript levels for 17 of the 29 *LACCASE* (*PtrLAC*) genes in the black cottonwood (*P. trichocarpa*) genome decreased in *P. trichocarpa* trees overexpressing miR397a, in turn leading to a reduction of lignin levels (Lu et al., 2013). Similarly, the overexpression of *miR319a* in *Populus tomentosa* in seedlings resulted in delayed secondary growth and decreased xylem production (Hou et al., 2020). In particular, *miR165b* guides the development of pith secondary cytoderm is by restraining the *AtHB15* (*HOMEOBOX 15*) expression domain (Du and Wang, 2015). However, how wood anatomical features like container shape and thickness are genetically governed is not very clear. These traits are important for cell wall composition and overall tree performance (Quan et al., 2018; Quan et al., 2019).

To explore the molecular basis of changes in transcript levels caused by miRNAs during wood formation, we sequenced small RNAs from three wood parts: normal wood (NW)which is from the stem of the tree at breast height, tension wood (TW) which is upper side of the bending trunk, and opposite wood (OW) which is lower side of the bending trunk. We then assigned predicted target genes to these wood-abundant miRNAs by identifying genes whose transcript levels were inversely correlated with miRNA abundance. We complemented this approach by using a bioinformatics method for miRNA target prediction and constructed the resulting miRNA–mRNA interaction network. The small RNAs found in this study are good candidates for miRNAs that are involved in wood formation. They may also help with the development of functional markers for molecular breeding in rubber trees and other tropical plants to help change the composition of lignin or physical characteristics.

## Materials and methods

### Plant materials and microscopy observations

The rubber trees (clone Reyan 7-33-97) used in this study were grown in the experimental greenhouse of Hainan University (Danzhou, Hainan, China; 109°29′25′′ E, 19°30′40′′ N) at the end of June 2016. To probe the genes involved in the formation of reaction wood, three rubber trees of similar age and with trunks of similar diameter (about 2 cm) were bent at a 30° angle for 300 days and were selected as experimental materials to force the formation of reaction wood (**Figure S1**). The bending was applied starting at 9 a.m. on August 17th, 2020, and ended at 9 a.m. on June 13rd, 2021, at which point the wood samples were rapidly processed. The wood quality from the collected samples was assessed by scanning electron microscopy (SEM, Phenom proX, the Netherlands) in Center for Analytical Instrumentation (Hainan University), and the resulting images were processed in ImageJ software to measure the area of the gelatinous (G) layer. The SEM images indicated that the TW (tension wood) reaction wood had an extremely dense cementitious layer (G-layer; **Figure S2A**) that was not present in NW (normal wood) or OW (opposite wood; **Figures S2B, C**). These observations were consistent with earlier findings (Sujan et al., 2015). Therefore, xylem samples from the collected trees were selected for further analysis.

Samples were collected for NW, TW, and OW from the same individuals to allow for direct comparison in an identical genetic background. Briefly, the bark above the sampling area was removed to expose the inner wood layers. A sharp razor blade was then used to collect TW (upper side) and OW (lower side) from the same branch (Li et al., 2013). The control for stem xylem tissue was NW and was collected about 1 m above the ground, before the bending point. All collected samples were about 2 cm × 1 cm and 4–5 mm in depth. Samples were harvested in the morning, quickly frozen in liquid nitrogen, and stored at –80°C until use.

### RNA extraction and qualification

Total RNA was extracted from nine samples (NW1, NW2, NW3, OW1, OW2, OW3, TW1, TW2, and TW3) using a modified cetyl trimethyl ammonium bromide (CTAB) method (Chang et al., 1993). Each tree counted as one biological replicate (NW1-3, TW1-3, and OW1-3). Traces of genomic DNA were removed with RNase-free DNase I digestion (Takara, Beijing, China). RNA degradation and DNA contamination were assessed by electrophoresis on 1% (w/v) agarose gels. RNA concentration, quality, and integrity were estimated on a NanoPhotometer spectrophotometer (IMPLEN, CA, USA) and an RNA Nano 6000 Chip on a Bioanalyzer 2100 (Agilent Technologies, CA, USA). Only RNA samples with an OD_260/280_ ratio of 1.9–2.2, an OD_260/230_ ratio ≥ 2.0, and RNA integrity number (RIN) values > 6.8 were processed for further experiments.

### Small RNA library construction and sequencing

Three micrograms of total RNA per sample was used to construct sequencing libraries with a NEBNext multiplex small RNA library prep kit for Illumina (NEB, USA) following the manufacturer’s protocol. Briefly, the NEB 3′ SR adapter was ligated to the 3′ end of miRNAs, siRNAs (small interfering RNAs), and piRNAs (piwi-interacting RNAs). The SR real-time primer was then annealed to the 3′ SR adapter to initiate double-stranded DNA (dsDNA). The 5′ end adapter was connected to the 5′ ends of miRNAs. First-strand cDNA synthesis with M-MuLV Reverse Transcriptase (RNase H-). The libraries were amplified by 35 PCR cycles with index (X) primer, SR primer for Illumina, and LongAmp Taq 2X master mix. The PCR products were separated on 8% polyacrylamide gel for 80 min at 100 V. DNA fragments responding to 140-160 bp (the length of sRNAs with 5′ and 3′ adapters) were purified from the gel and eluted in 8 µL of elution buffer. Library quality and titer were evaluated using a DNA high sensitivity chip and Agilent Bioanalyzer 2100 instrument. A TruSeq SR Cluster Kit v3-cBot-HS (Illumina) was used for cluster formation on a cBot cluster generation system following the manufacturer’s instructions. Libraries were sequenced on an Illumina HiSeq 2,500 platform as 50-bp single-end reads.

### Identification of known miRNAs and novel miRNAs

After adapter trimming from the raw reads, any clean reads shorter than 18 nt or low-quality reads (reads with N bases > 10% and reads with a 3′ end with Q < 20 [Q = –10Log_10_^error_ratio^]) were discarded. The remaining clean reads were mapped to the rubber tree reference genome (Liu et al., 2020) with Bowtie2 allowing no mismatch (Langmead et al., 2009). Reads derived from rRNAs, protein-coding genes, snRNAs, tRNAs, snoRNAs, and repeat sequences were removed by filtering the clean reads against the Rfam database and RepeatMasker (Tempel, 2012; Kalvari et al., 2018). Known miRNAs were identified with miRBase 20.0 (Meng et al., 2018); potential miRNAs and their secondary structures were determined using the miRDeep2 algorithm (Friedländer et al., 2012) and sRNA-tools-cli (http://srna-workbench.cmp.uea.ac.uk/). The formation of a typical hairpin structure was used as a criterion to identify novel miRNAs from the transcripts showing no match to known miRNAs. Unannotated small RNAs were assessed with miRDeep2 (Friedländer et al., 2012) and miREvo (Wen et al., 2012) using minimum free energy, possible Dicer cleavage sites, and the secondary structures of small RNA tags. Custom scripts were applied to count all candidate miRNAs and estimate the basic deviation between each position of all identified miRNAs and the first position of identified miRNAs of a given length. The secondary structure of the novel_28 mature sequence was predicted and compared to other miRNA families from rubber tree and other species.

### Prediction of the target genes of miRNAs

The RNA-seq data were from our previous study (Meng et al., 2021). The online tool psRNATarget (https://www.zhaolab.org/psRNATarget/) was used to predict miRNA targets with default parameters with expectation ≤ 3 (Dai et al., 2018). A gene was deemed to be a putative target for a miRNA when its transcript levels showed a negative Pearson’s correlation coefficient with miRNA abundance (correlation < –0.8, *P*-value < 0.05). miRNA abundance was estimated with the formula (Zhou et al., 2010): Normalized abundance = mapped read count/total reads * 1,000,000.

### KEGG enrichment analysis of co-expressed target genes

The predicted co-expressed target genes were subjected to KEGG (Kyoto Encyclopedia of Genes and Genomes) pathway. KEGG (http://www.genome.jp/kegg/) pathway enrichment analysis was carried out with KOBAS software (Mao et al., 2005).

### Construction for miRNA–mRNA interaction networks and tissue-specific expression analysis

The Pearson’s correlation coefficients between miRNAs and transcription factor genes were calculated using expression values from this study for miRNAs and from our previous study (Meng et al., 2021) for mRNAs or between transcription factor genes and other genes to identify miRNAs, transcription factor genes, and their co-expressed genes. The resulting interaction network was built in R (version 4.0.1) and visualized with Cytoscape (version 3.8.1).

### Plasmid construction

**Figure 1:**
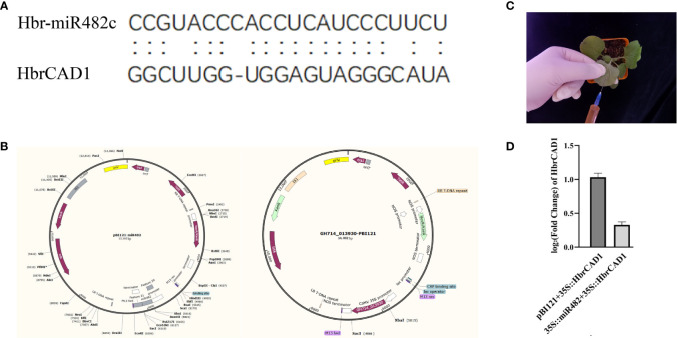
Validation of the targeting of *HbrCAD1* by its predicted miRNA novel_28 in *Nicotiana benthamiana*. *β-actin* was selected as internal reference; data are means ± standard error of three independent biological replicates. **(A)** Alignment of *hbr-miR482c* and *HbrCAD1*. **(B)** The plasmids pBI121-miR482c and GH714-013930-pBI121 used for the assay. **(C)** Principle of transient infiltration of *N. benthamiana* leaves with an Agrobacterium cell suspension. **(D)** Relative *HbrCAD1* expression levels in different samples. Data are shown as Log2 (fold-change), with the expression of *HbrCAD1* from the pair pBI121 + *35S:HbrCAD1* set to 1.

According to the target sites (**Figure 1A**), to overexpress novel_28 (*hbr-miR482c*), the *pre-miR482c* sequence was amplified from genomic DNA isolated from normal wood tree with primers containing BamH I and Sac I restriction sites. The resulting PCR product was digested with BamH I and Sac I and ligated into the vector pBI121 downstream of the cauliflower mosaic virus (CaMV) 35S promoter (Shanghai Generay Biotech; **Figure 1B**). The full-length coding sequence of *HbrCAD1* (GH714_013930) was also cloned into pBI121 (GH714_013930-pBI121; **Figure 1B**). Both constructs were transformed into *Agrobacterium* (*Agrobacterium tumefaciens*) strain GV3101. The primer sequences used for cloning are listed in **Table S1**.

### Verification of interaction between *hbr-miR482c* and its target

Agrobacterium-mediated transient expression in *Nicotiana benthamiana* leaves (English, 1997) was used to assess the targeting of *HbrCAD1* transcripts by hbr-miR482c. Agrobacterium cultures harboring the *hbr-miR482c* or HbrCAD1 construct were resuspended in infiltration buffer, mixed, and infiltrated into six *N. benthamiana* leaves (**Figure 1C**) from 3-week-old plants. As negative controls, Agrobacterium cultures a mix of cultures harboring pB121 and the 35S::HbrCAD1 construct were infiltrated in N. benthamiana leaves.

### Validation of miRNA expression and that of their target genes by RT-qPCR

A GoScript™ Reverse Transcription kit (Promega, USA) was used for first-strand cDNA synthesis from total RNA of the nine samples collected in this study. Six differentially expressed miRNAs (novel_47, novel_67, novel_28, novel_165, novel_177, and novel_101) and six target genes (*ALDO1*, *PEPcase*, *CAD1*, *RF2*, *PKc_like*, *GT*) of miRNA-mRNA correlation network were chosen (**Figure S3**). Gene-specific primers were designed for the target genes with Primer Premier v5 software (**Table S1**). qPCR was performed with TB Green Premix Ex Taq II (Tli RNase H Plus; Takara, Beijing, China) for the target genes according to the manufacturer’s instructions. PCR conditions were as follows: denaturation at 94°C for 2 min, then 40 cycles of 95°C for 5 s and 60°C for 30 s. *Ubiquitin* was used as internal reference for normalization of expression data of rubber tree samples (Meng et al., 2022), and *β-actin* was used for *N. benthamiana* (Nawaz et al., 2019). For miRNA, a miRNA RT-qPCR Detection Kit (Aidlab, Beijing, China) was used following the manufacturer’s instructions. PCR conditions were as above. *U6* transcripts were used as internal reference for normalization (Zeng et al., 2009). A melting curve was performed from 60°C to 95°C to confirm the specificity of the amplicons. Relative expression levels of miRNAs and their target genes were estimated by the 2^–△△Ct^ method (Schmittgen and Livak, 2008). Three technical replicates were analyzed per sample.

## Results

### Overview of small RNA sequencing from reaction wood

We constructed nine small RNA libraries from different wood tissues (NW, TW, and OW) to explore the role of miRNAs in wood development in rubber trees. We obtained between 10.15 and 14.50 million raw reads after sequencing. We removed low-quality reads and removed adapters to yield 9.86 to 14.35 million clean reads with a length ranging from 18 to 30 nucleotides (nt) (**Table 1**).

**Table 1:** Summary of reads from small RNA sequencing.

Sample	Raw reads	Clean reads	Total sRNA	Mapped sRNA	Mapping ratio (%)
NW1	10,891,608	10,601,788	8,002,438	7,378,835	92.21%
NW2	14,495,357	14,353,830	11,160,172	9,992,003	89.53%
NW3	12,182,526	11,943,571	9,115,657	8,359,523	91.71%
TW1	10,151,849	9,858,994	6,329,046	5,936,751	93.80%
TW2	12,222,618	11,860,937	8,982,250	8,115,462	90.35%
TW3	13,032,187	12,697,455	9,391,865	8,629,289	91.88%
OW1	13,364,645	12,973,816	10,208,783	9,422,696	92.30%
OW2	11,209,112	10,839,323	6,835,678	6,268,583	91.70%
OW3	12,370,905	12,040,308	8,412,275	7,664,797	91.11%

**Figure 2:**
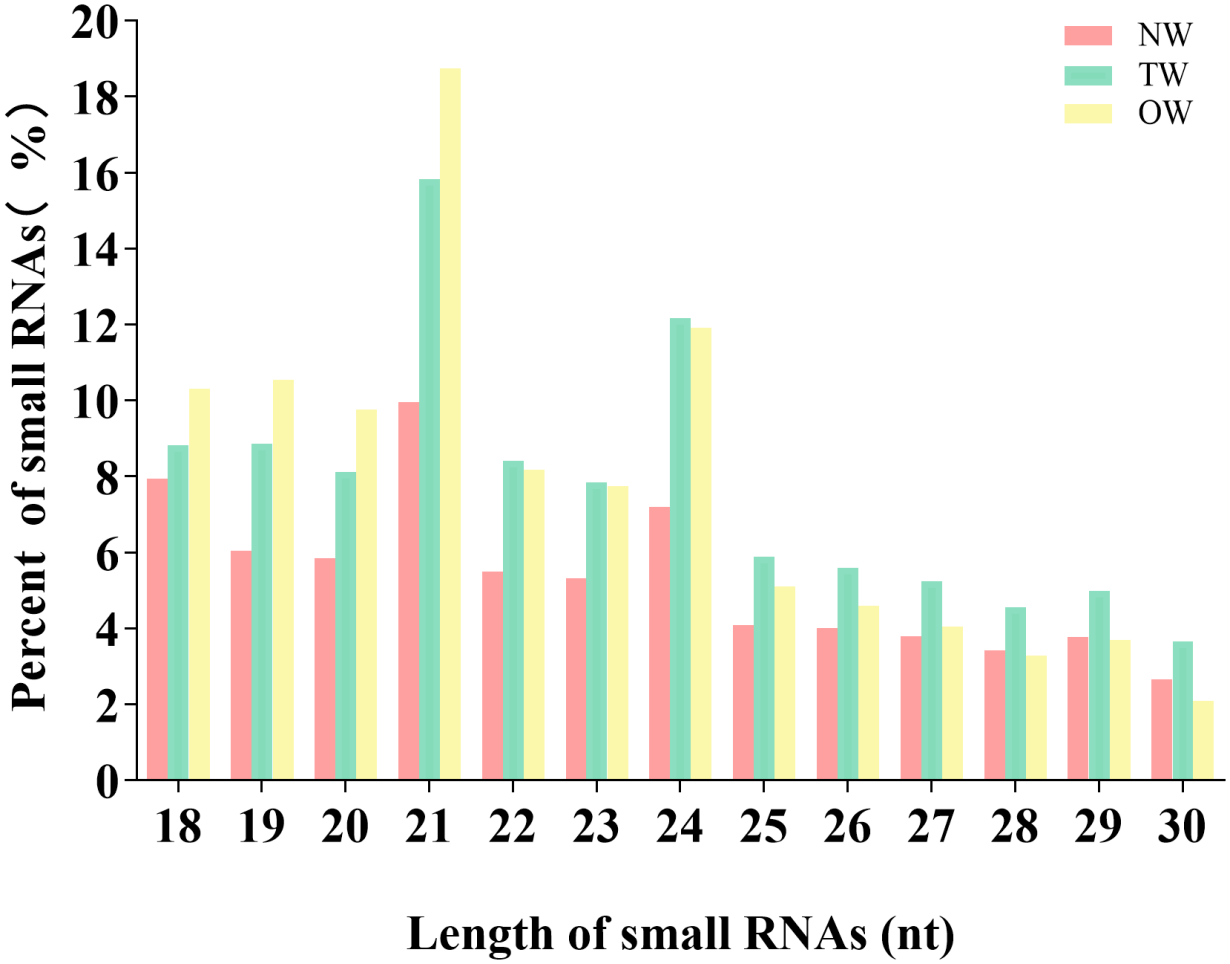
Length distribution of miRNA sequences sequenced from *Hevea brasiliensis* reaction wood.

We aligned the clean reads against the rubber tree reference genome with Bowtie2 and used RSEM software to estimate read counts per gene model. We successfully mapped from 5,936,751 to 9,992,003 reads to the rubber tree genome, or 89.53%-93.80% of all clean reads across the nine small RNA libraries (**Table 1**). We then removed all reads mapping to tRNA (transfer RNA), snoRNA (small nucleolar RNA) and snRNA (small nuclear RNA) loci. We focused on the remaining unannotated reads. We observed a peak for 21-nt sRNAs, representing >14% of all candidates unannotated sRNAs in the libraries, with a second peak for 24-nt sRNAs (from 10% to 13%; **Figure 2**).

### Annotation of known and novel miRNAs expressed in reaction wood

**Figure 3:**
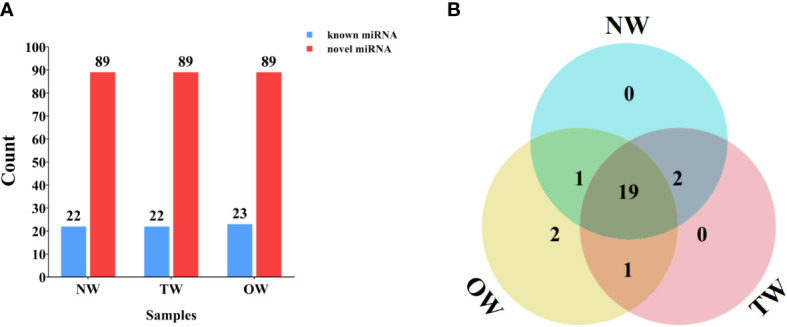
Numbers of miRNAs identified in different xylem tissues. **(A)** Known and novel miRNAs identified from small RNA sequencing of NW, TW, and OW from 300-day rubber tree reaction wood. **(B)** Venn diagram showing the extent of overlap between known miRNAs in each tissue type (NW, TW, and OW) from 300-day rubber tree reaction wood.

We compared the unannotated sRNAs identified above to all miRNAs or their *pre-miRNA*s deposited in miRBase to identify known and novel miRNAs. We identified 22 (NW), 22 (TW), and 23 (OW) known miRNAs belonging to 21 miRNA families (**Figure 3A**). Of these, 19 miRNAs were shared across the NW, TW, and OW libraries (**Figure 3B**; **Table S2**). We determined that the first base of these known miRNAs tended to be a uracil (U) for 18–22-nt miRNAs (**Figure S4**).

The known miRNA *hbr-miR166*, *hbr-miR396*, *hbr-miR408*, and *hbr-miR482* families were each represented by two members, while the remaining miRNA families only had one member (**Figure S5**). Furthermore, two miRNAs (*hbr-miR6170* and *hbr-miR6171*) were specific to OW (**Figure S5**), suggesting that each wood tissue is associated with a slightly different set of miRNAs.

The sequences failed to match known miRNAs or *pre-miRNA*s were used to predict novel miRNA resulting in 89 novel miRNAs with typical hairpin structure (**Figure 3**, **S6**). The first base of these novel rubber tree miRNAs (18–30 nt in length) also largely started with a uracil (**Figure S7**). Furthermore, we cloned and assigned novel_28 to the miR482 family through BLAST search against other species in the miRBase database. We then predicted the secondary structure of novel_28 and compared its mature sequence to that of other *hbr-miR482* family members, which revealed novel_28 is a new member of the *hbr-miR482* family (**Figures S6**, **S8**). Thus, we renamed novel_28 as hbr-miR482c.

### Differentially abundant miRNAs

To investigate how miRNAs might regulate wood formation, we quantified the abundance of all miRNAs as TPMs (transcripts per million), followed by a comparison of miRNA abundance between the tree wood types (|Fold change|≥2 and *P* ≤ 0.05). The OW vs. NW comparison yielded 11 differentially abundant miRNAs, 5 for the TW vs. NW comparison, and 2 for the TW vs. OW comparison. Of the five differentially abundant miRNAs between TW and NW samples, three were more abundant in TW and two were more abundant in NW tissues. Similarly, 5 miRNAs were more accumulated in OW and 6 were more accumulated in NW tissues. Finally, one miRNA was more abundant in each TW and OW tissue (**Table S3**).

### Prediction of miRNA target genes

We predicted miRNA targets through psRNATarget (Dai et al., 2018), which identifies transcripts with complementary sequences to those of miRNA candidates. We independently calculated Pearson’s correlation coefficients between the abundance of each miRNA and the transcript levels of all rubber tree transcripts obtained from a previous study (Meng et al., 2021). We considered genes as candidate targets when their transcript levels were negatively correlated (<–0.8) with miRNA abundance. In the TW vs. NW comparison, we determined that 4 novel miRNAs have the potential to target 10 genes, but no clear target could be identified for the novel_86 miRNA. Similarly, we identified 31 targets for 8 of the differentially abundant miRNAs in the OW vs. NW comparison, with no predicted targets for the other three novel miRNAs (**Table S4**). Further, we identified 11 target genes for the 2 differentially abundant miRNAs from the comparison between TW and OW. We selected those potential targets with a negative correlation of –0.8 or below with their associated miRNA, resulting in 39 putative targets for 12 novel miRNAs (cor ≤ –0.8 and *P* ≤ 0.05) (**Table S5**).

### Identification of genes co-expressed with transcription factor genes involved in rubber tree reaction wood formation

In the above list of target genes, we noticed that 53.85% (or 21 of 39) encode transcription factors (TFs; **Figure S9**). To define the putative targets of the encoded TFs, we measured the Pearson’s correlation coefficients (PCCs) between TF genes and all other rubber tree genes, using RNA-seq data available at the NCGC (National Genomics Data Center) under the accession numbers CRA004241 and CRA004243. A KEGG pathway enrichment analysis showed that these co-expressed targets are largely related to phenylpropanoid biosynthesis, phenylalanine metabolism, and fatty acid biosynthesis (**Figure S10**). These results suggest a central role for these TFs during reaction wood formation under prolonged mechanical stress.

### Differentially expressed mRNA-miRNA pairs related to wood formation

**Figure 4:**
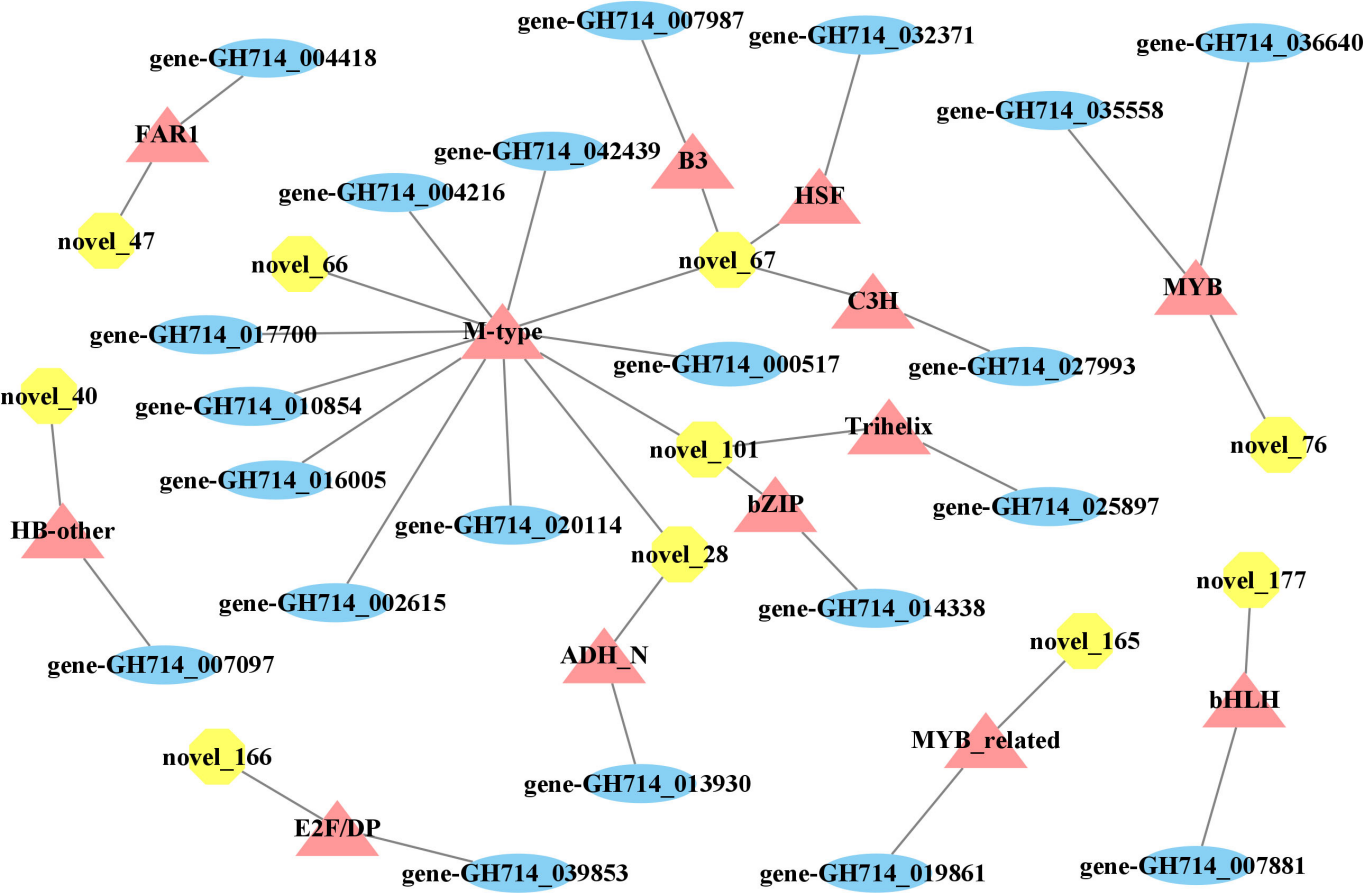
miRNA-transcription factor-mRNA networks associated with wood formation. Yellow octagons represent miRNAs, orange triangles represent transcription factor genes, and blue ellipses represent genes.

**Figure 5:**
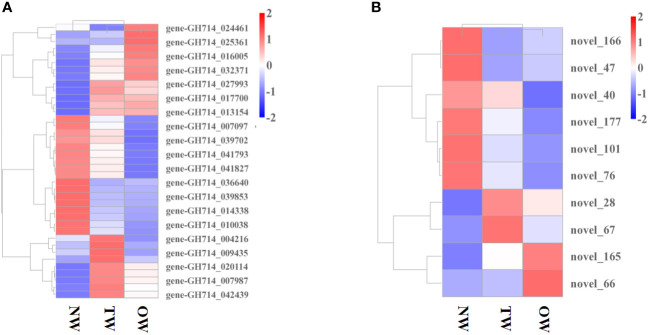
Expression profile of miRNAs and genes co-expressed with transcription factor genes. **(A)** Heatmap representation of the expression levels of genes co-expressed with transcription factor genes in different tissues. **(B)** Heatmap representation of the expression profile of miRNAs in TW, OW, and NW. Columns represent three different tissue types and rows represent different transcripts. Each square represents a transcript and the color indicates the level of expression; red represents up-regulation and blue represents down-regulation.

We then explored how miRNA-mediated adjustment of transcripts affected reaction wood development. Here, we focused on target genes co-expressed with those TF genes that were associated with phenylalanine metabolism or carotenoid biosynthesis and constructed the underlying interaction network (**Figure S10**, **4**). Our above analysis predicted that *hbr-miR482c* modulates the transcript levels of *HbrCAD1*, which is related to phenylpropanoid biosynthesis. Similarly, novel_67 might modulate the transcript levels of genes related to lignin biosynthesis by targeting C3H-type zinc finger TF family members (**Figure 4**); novel_93 was predicted to modulate the transcript levels of genes related to phenylalanine metabolism. In this network, *hbr-miR482c* and novel_76 each targeted two genes involved in wood formation. The C3H, FAR1 (FAR-RED IMPAIRED RESPONSE 1), bZIP, and MYB TF families possibly play vital functions in wood growth from our miRNA-TF-mRNA network. The target genes co-expressed with these TF genes, such as fatty acyl-CoA reductase 1 (*FAR1*, gene-GH714_004418), cinnamyl-alcohol dehydrogenase (*CAD1*), and p-coumarate 3-hydroxylase (*C3H*, gene-GH714_027993), were involved in carotenoid biosynthesis. We thus propose that the miRNA-TF-mRNA regulatory network described here may play a significant role in adjusting the molecular events necessary for reaction wood development in rubber tree. The expression levels of these genes and associated miRNAs are shown in **Figure 5**.

We wished to confirm the transcript levels of miRNAs and their target genes estimated from the RNA-seq datasets by a reverse transcription quantitative PCR (RT-qPCR) analysis of the same RNA samples. We observed a generally comparable trend in the accumulation of most miRNAs and their target genes between RT-qPCR and RNA-seq data in the OW, NW, and TW samples, although the fold-change (FC) values from RNA-seq did not precisely match the expression values obtained by RT-qPCR (**Figure S3**). These results indicate the dependability of the sequencing data.

### *HbrCAD1* transcripts are cleaved by *hbr-miR482c*

miRNAs can cause the cleavage of their target transcripts between the 10th and 11th nucleotides of their target site (Rhoades et al., 2002). In this study, we predicted from bioinformatics analysis that *hbr-miR482c* may influence lignin biosynthesis by targeting *HbrCAD1* transcripts (**Figure 4**). To test this hypothesis experimentally, we co-expressed the precursor of *hbr-miR482c* and HbrCAD1, driven by the strong cauliflower mosaic virus (35S) promoter, into *N. benthamiana* leaves by Agrobacterium-mediated transient infiltration. As negative controls, we co-infiltrated the construct expressing the *HbrCAD1* and the empty effector vector (pBI121). We observed that *HCAD1* transcript levels decrease significantly when the *hbr-miR482c* precursor is co-expressed (**Figure 1D**). Thus we suspected that *HbrCAD1* transcripts might be the target gene of *hbr-miR482c*.

## Discussion

### A model for reaction wood formation in rubber tree and other tree species

The scanning electron microscopy of the xylem samples from reaction wood had confirmed that the tension wood had the remarkably thick gelatinous layer (**Figure S2**). These results were supported by earlier findings and confirmed a tension wood model were constructed in our study (Sujan et al., 2015). We wished to identify genes important for wood formation based on their expression models in various wood tissues of rubber tree. We drew heatmaps and constructed an interaction network to reveal connections between genes related to phenylpropanoid biosynthesis.

Enriched KEGG terms underscored the involvement of phenylpropanoid biosynthesis, phenylalanine metabolism, and pyruvate metabolism in reaction wood formation. Indeed, our results showed that the genes co-expressed with miRNA-targeted TF genes were related to metabolism and physiological functions that all contribute to reaction wood development. Similar consequences have been reported in previous work, lending some support to our current study (Li et al., 2013; Lv et al., 2021). Future work should pay close attention to exploring the functions of noncoding RNAs and their candidate target genes predicted from our RNA-seq results. In particular, genes linked to wood development can now be identified from their expression patterns across wood growth regions.

### Integrated analysis of the miRNA-TF-mRNA network

Important transcription factors associated with secondary growth have been identified, such as members of the C3H and MYB TF families (Demura and Fukuda, 2007; Zhong and Ye, 2009). Zhang et al. reported that miRNAs may be connected to tension wood development by regulating secondary cell wall biosynthesis in Moso bamboo (*Phyllostachys edulis*) (Zhang et al., 2018). In this study, we determined that the transcript levels of 21 TF genes from 13 families changed over the course of wood bending (**Figure S9**). Of these, TF genes from the *C3H* and *MYB* families may help adjust the expression of genes related to phenylpropanoid biosynthesis and possibly play a significant role in the wood development in rubber tree. Previous research revealed that overexpressing the MYB TF genes *PtoMYB216*, *PtoMYB74*, and *PtoMYB92* from *P. tomentosa* induced the expression of genes related to lignin biosynthesis, resulting in thicker xylem cell walls, more xylem layers, ectopic lignin deposition, and enhanced lignin contents by 13–50% (Qiaoyan et al., 2013; Li et al., 2015; Li et al., 2018). Analogously, compared to the control, the expression levels of lignin biosynthetic genes, lignification ability, xylem volume, and lignin levels of *C3H* overexpressed in *Arabidopsis* all increased (Fornalé et al., 2015). We also observed that C3H-type zinc finger TF family members possibly take part in wood development by regulating the transcript levels of *GLUCOSYLTRANSFERASE* (*GT*) (gene-GH714_027993), which is associated with cellulose biosynthesis. This observation underscores the significance of *C3H* family members during plant development.

### miRNA regulaged key genes in wood formation pathway

The proteins that are thought to catalyze glucan-chain elongation in cellulose and callose biosynthesis are processive GTs that belong to GT2 family. In *Arabidopsis*, 10 to 12 GT2 family members form CESA (cellulose synthase catalytic subunit) and callose synthase (Yang et al., 2016). Moreover, GTs are a vital component of wood development. For instance, in *Arabidopsis*, CesA8 participates in secondary cell wall formation, as its loss-of-function mutation produced plants with a delicate stem phenotype (Taylor et al., 1999; Taylor et al., 2000; Taylor et al., 2003). Similarly, a mutation in rice *CesA8* caused a dramatic decrease in the cellulose content of secondary cell walls, resulting in a brittle culm phenotype (Zhang et al., 2009; Song et al., 2013). Meng et al. found that *HbrCesA8*, which is related to cellulose biosynthesis, may participate in reaction wood formation in rubber tree (Meng et al., 2021). These findings support our miRNA-mRNA model for rubber tree wood formation. Our multiomics-based approach produced a post transcription network of which several nodes are regulated by novel_67, which possibly affects some target genes during wood development.

The expression levels of *CAD*, *4CL*, peroxidase (*POD*), and *CAFFEATE O-METHYLTRANSFERASE* (*COMT*) are specifically linked to lignin composition (Do et al., 2007; Wagner et al., 2009; Vanholme et al., 2010; Chanoca et al., 2019). Here, we showed that *HbrCAD1* transcript levels are less abundant in OW tissues compared to NW, suggesting that HbrCAD1-catalyzed reactions might contribute less to wood formation in OW relative to NW tissues. Previous studies have shown that repressing *PAL*, *CAD*, or other enzymes of the lignin biosynthetic pathway may cause decreased lignin content (Chanoca et al., 2019). Furthermore, in *Arabidopsis*, studies have indicated that single or double knockout mutants in *POD*s representatively resulted in a small but significant decrease in lignin content and altered lignin biosynthesis in inflorescence stalks, indicating that modulating *POD* expression levels may affect lignin composition (Herrero et al., 2013; Barros et al., 2015). In general, we hypothesize that the expression pattern of *HbrCAD1* contributes to establishing the difference in lignin levels across tissue types.

## Conclusions

Based on the small RNA data obtained from wood tissues collected from rubber tree, we identified 114 miRNAs (25 known and 89 novel) present in 300-day reaction wood. We also established a network linking miRNAs, their putative TF target genes, and the genes that are co-expressed with these TF genes in the context of cellulose biosynthesis. Finally, we revealed the interaction landscape of these three regulatory layers in adjusting reaction wood growth and validated the network in wood formation of rubber trees. In summary, we described target genes associated with wood development in rubber tree and studied their post-transcriptional regulation. These results will provide the theoretical basis to clarify miRNA-mediated post-transcriptional mechanisms during wood growth and development in rubber trees.

## Data availability statement

The datasets presented in this study can be found in online repositories. The names of the repository/repositories and accession number(s) can be found below: https://bigd.big.ac.cn/gsa, CRA007612 https://bigd.big.ac.cn/gsa, CRA004818.

## Author contributions

JHC designed the research. MML, XM, YZ, YW, NJ and JMC performed the research. All authors analyzed and interpreted the data. JHC and MML wrote the manuscript. All authors contributed to the article and approved the submitted version.
