# Supplementary material for: Multiomics studies with co-transformation reveal microRNAs via miRNA-TF-mRNA network participating in wood formation in Hevea brasiliensis
Source: Front Plant Sci. 2023 Aug 14;14:1068796. doi: 10.3389/fpls.2023.1068796 (PMC10461101; doi:10.3389/fpls.2023.1068796)
Supplement: Supplementary file 1 [file DataSheet_1.pdf]

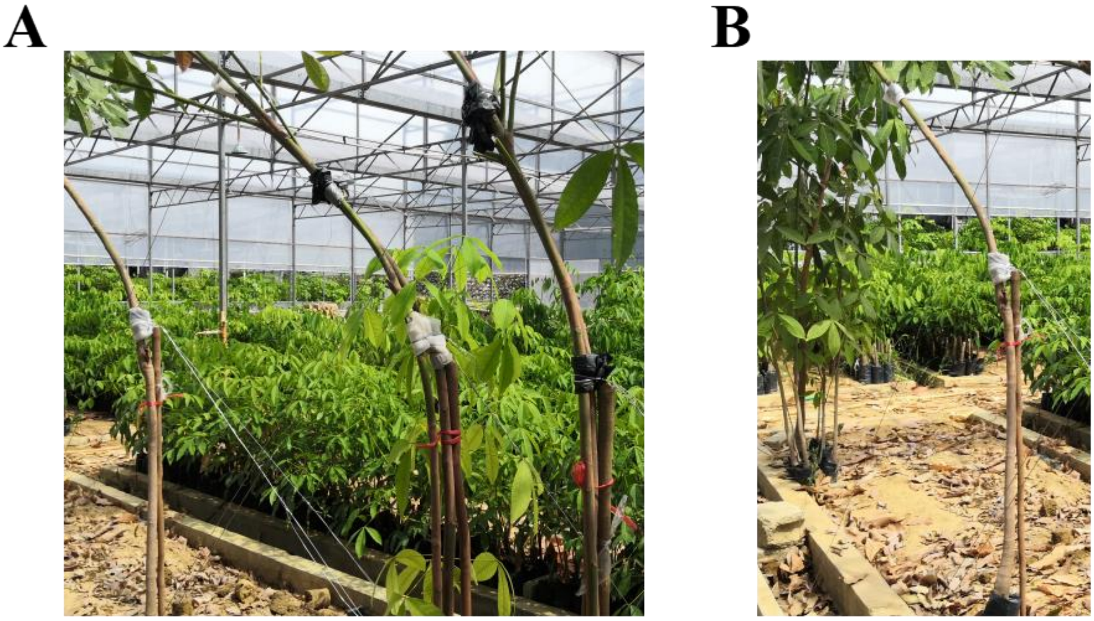

Figure S1. Representative photographs of rubber trees bent at a consistent angle of 30° for 300 days to induce the formation of reaction wood. (A) Photograph of the three independent biological samples. (B) Photograph of one independent biological sample.

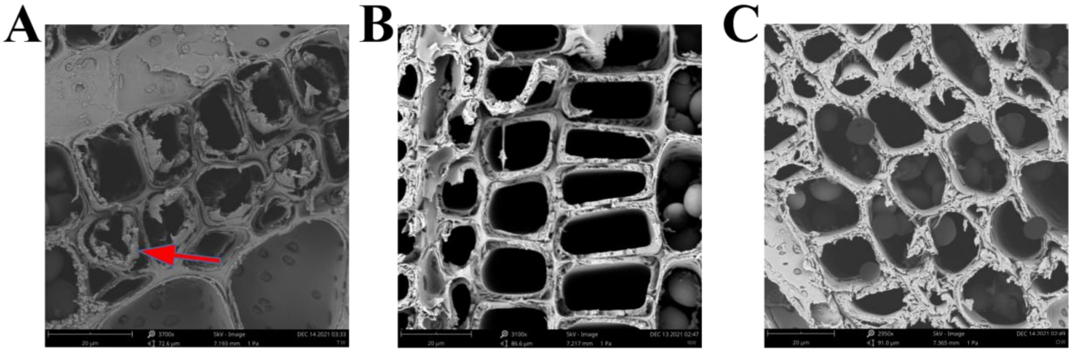

Figure S2. Microscopic structure and Runkel ratio of 300-day rubber tree reaction wood used in this study. Microscopic structure of tension wood (A), normal wood (B), and opposite wood (C) from 300-day rubber tree reaction wood. The red arrow indicates the G-layer.

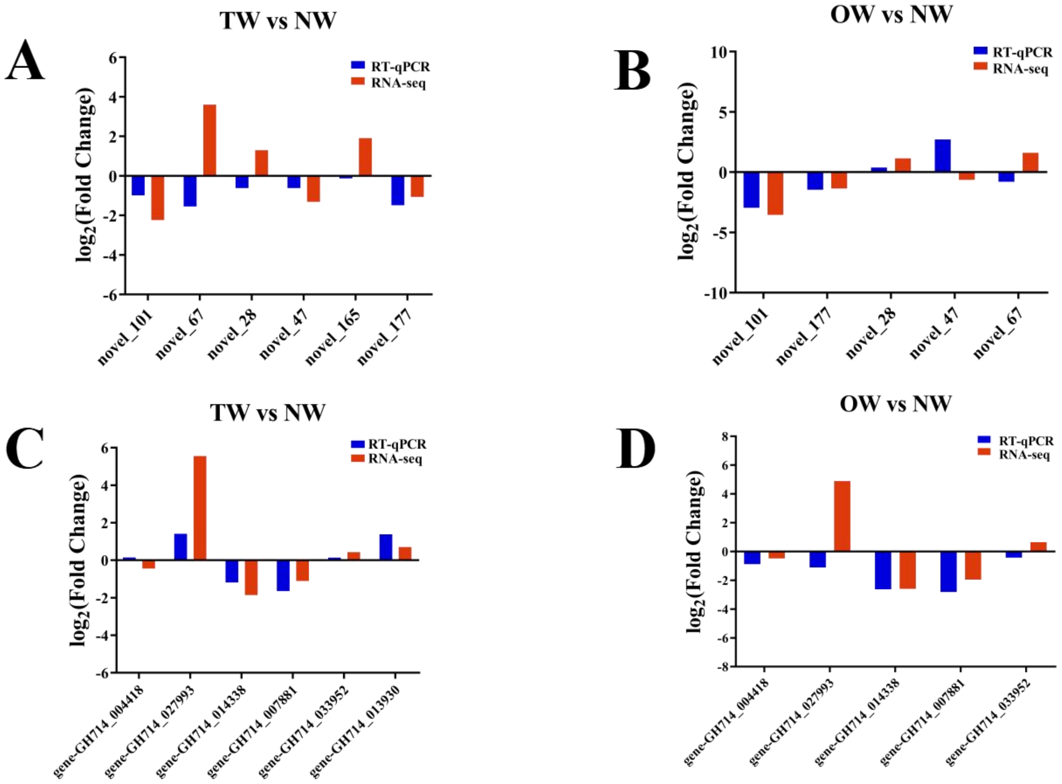

Figure S3. RT-qPCR validation of the expression levels of miRNAs and genes in NW, TW, and OW tissues from 300-day rubber tree reaction wood. *Ubiquitin* and *U6* transcript levels were used as internal references for mRNAs and miRNAs, respectively.

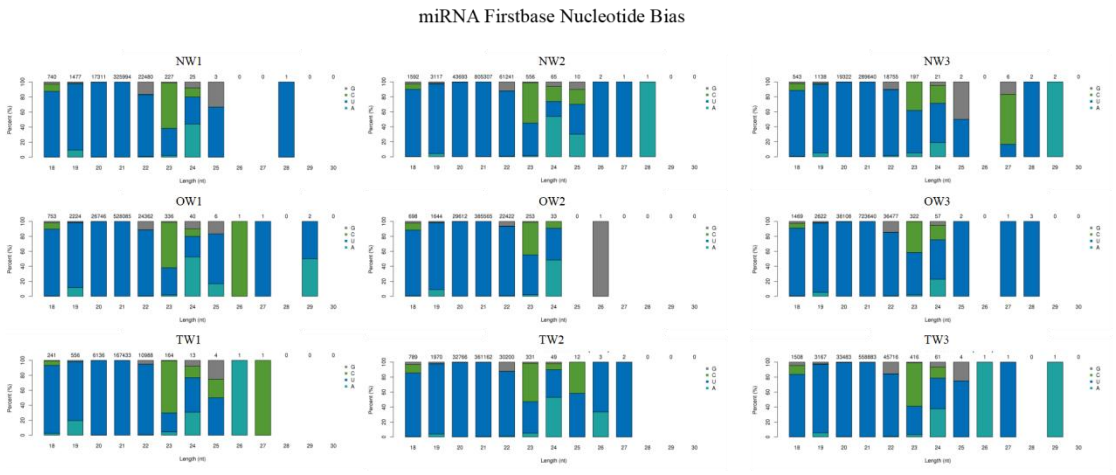

Figure S4. Sequence frequency of the first base in known miRNAs from 300-day wood samples with lengths of 18–30 nt.

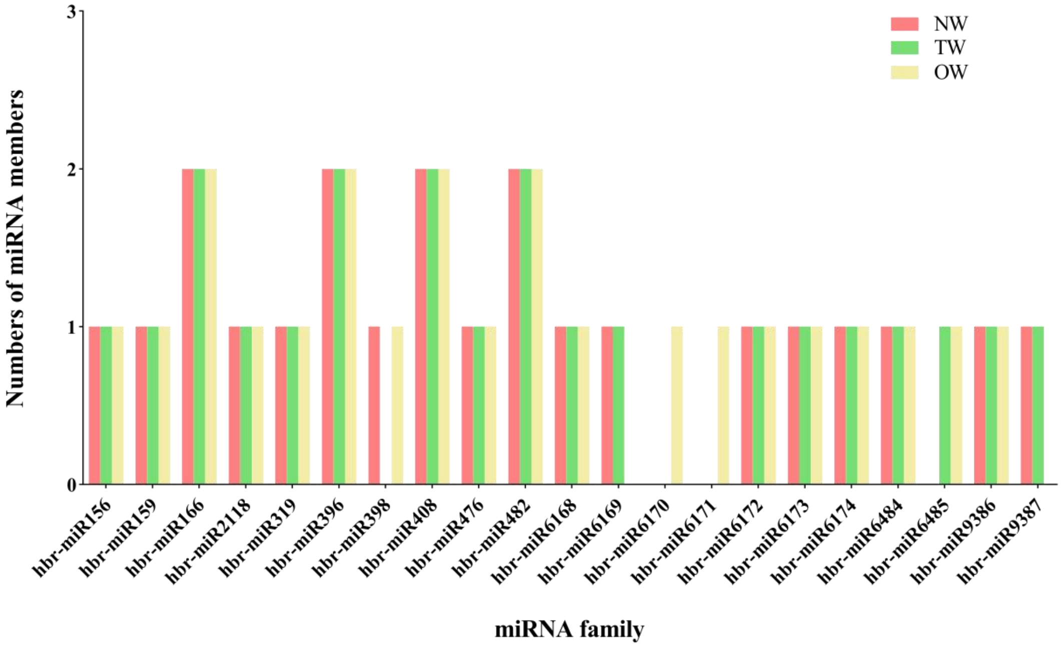

Figure S5. Identification of miRNA families in 300-day rubber tree reaction wood.

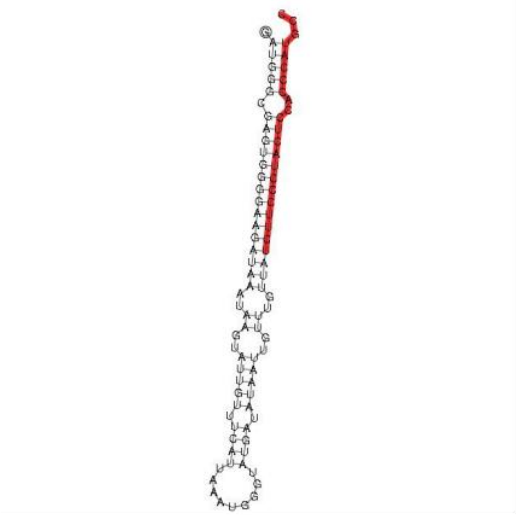

novel_28

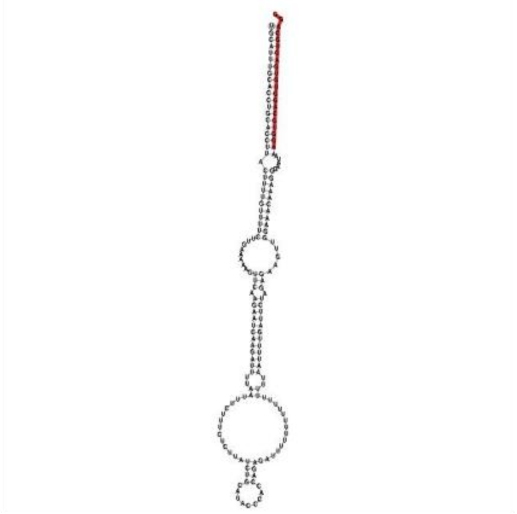

novel_67

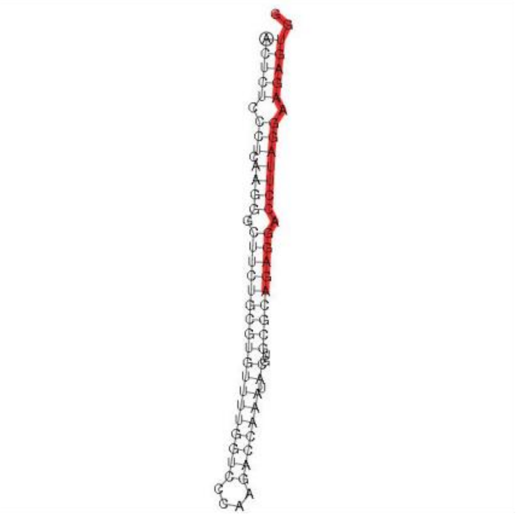

novel_93

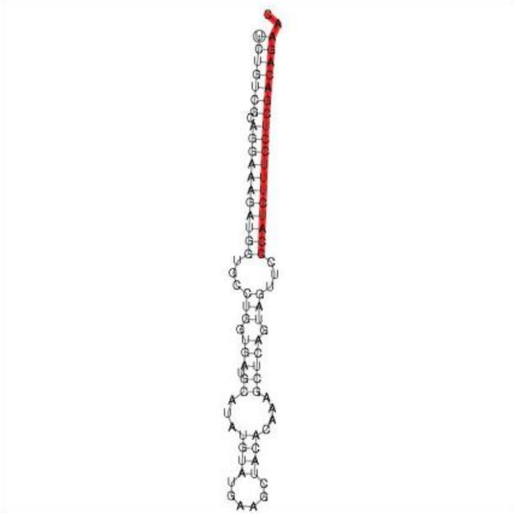

novel_165

Figure S6. Prediction of the secondary structure of novel miRNAs in 300-day wood samples.

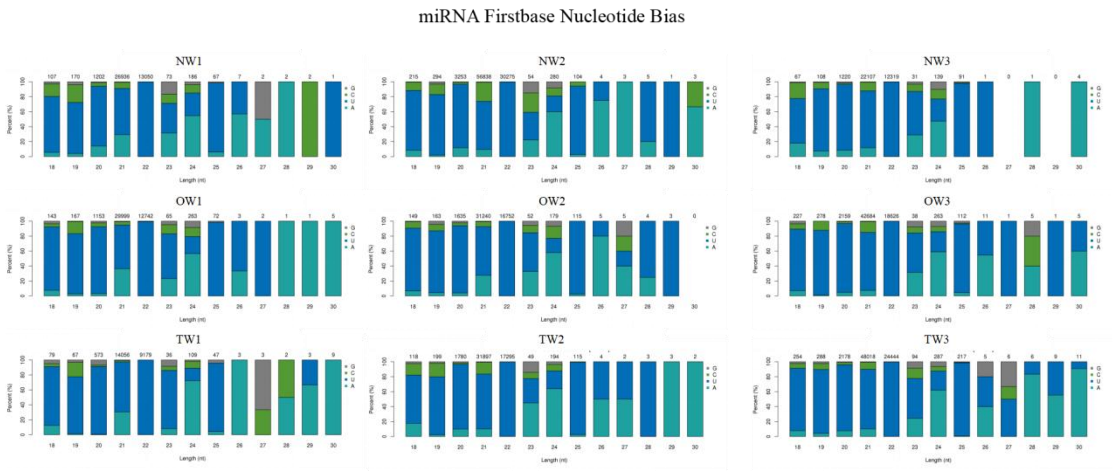

Figure S7. Sequence frequency of the first base of novel miRNAs from 300-day wood samples with lengths of 18–30 nt.

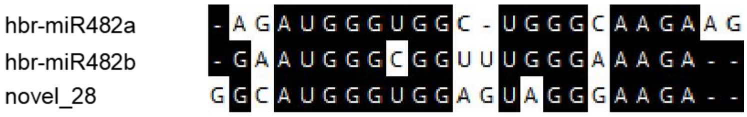

Figure S8. Multiple sequence alignment between novel_28 and the hbr-miR482 family.

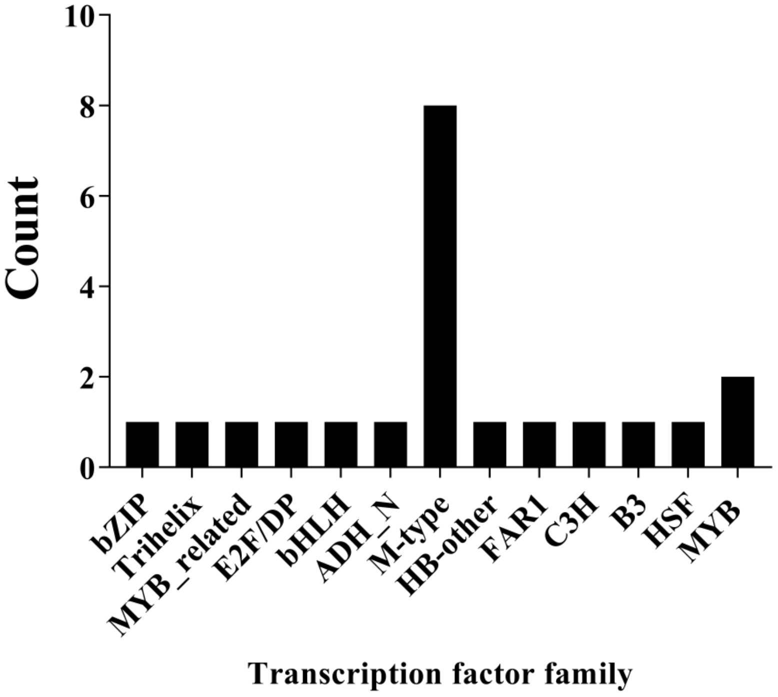

Figure S9. Number of transcription factors among miRNA target genes from 300-day rubber tree reaction wood, sorted by family.

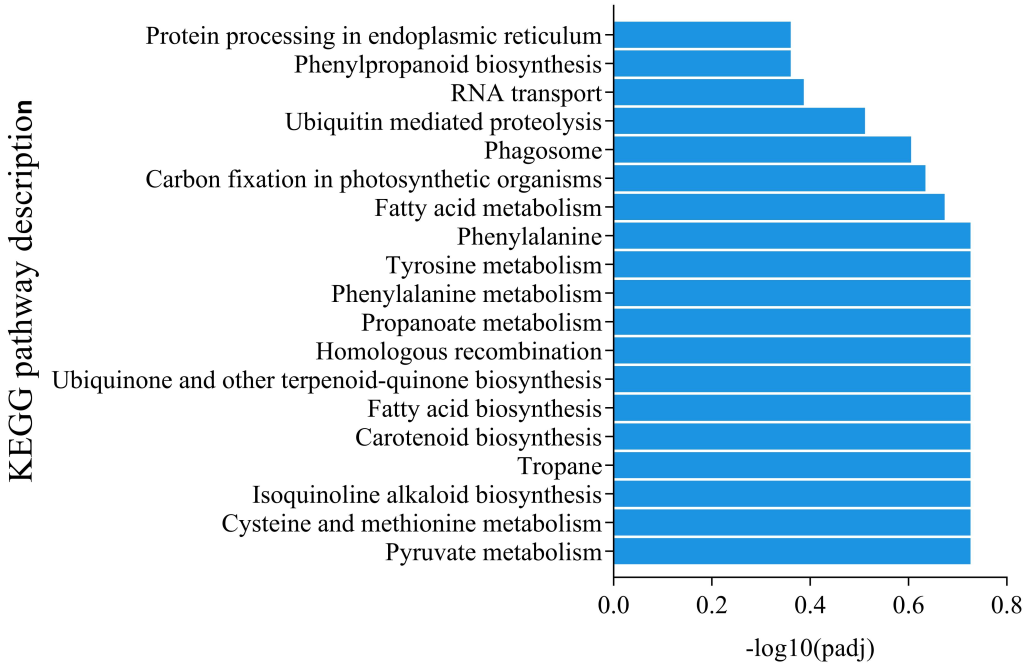

Figure S10. Functional analysis of the genes co-expressed with transcription factor genes. KEGG enrichment analysis of genes co-expressed with transcription factor genes in 300-day rubber tree.
