# Supplementary material for: Multiomics studies with co-transformation reveal microRNAs via miRNA-TF-mRNA network participating in wood formation in Hevea brasiliensis
Source: Front Plant Sci. 2023 Aug 14;14:1068796. doi: 10.3389/fpls.2023.1068796 (PMC10461101; doi:10.3389/fpls.2023.1068796)
Supplement: Supplementary file 2 [file DataSheet_2.pdf]

Table S1. Oligonucleotide primers used for qPCR and plasmid construction in this study.

Gene name	Forward primer	Reverse primer
Ubiquitin	ATGCAGCACCGGGAATAAGT	GGTCATCAGGGTTTGGAGCA
ß-actin	CTATGTTCCCTGGTATTGCAGACC	GCCACAACCTTGATTTTCATGC
U6	CATCCGATAAAATTGGAACG	GATTTGTGCGTGTCATCCTT
ALDO1	TGAAGGATGAAGGGATTG	TTCTAGCATAGTCTAGGGAGT
PEPcase	CATACAGGCGAAGGATTA	AGCATACAACTGGGTCAA
CAD1	CCTTATACCTACACGCTCAG	ATCCCACCTCCACTACCT
RF2	AAAGTGAAACGGGTAAGA	CATGAACTCGCTGTAAAA
PKc_like	CCGATTCTATTTCCATTC	AGATACCTTGCCACCTAA
GT	TTTGCCTCGGTTGAGTTG	ATTGCTGCTTTGGTCCCT
novel_47	ACTCTCCCTCAGGGCTTCAAG	
novel_67	AGGTGCAGGTGTGAGTGCA	
novel_28	TCTTCCCTACTCCACCCATG	
novel_165	GCCCATCTTTCCTCGACAGAAC	
novel_177	TGAAGCTGCCAGCATGATCTA	
novel_101	AGATCATGTGGCAGTTTCACC	
CAD1-OE	ATACCTACACGCTCAGAAA	ATCCCACCTCCACTACCT
miR482c-OE	GCGCACTATATAAGTGAGGCC	CTAAGACGTATACATGGCGG

Table S2. Known miRNAs identified from 300-day reaction wood.

miRNAs in NW3	miRNAs in OW3	miRNAs in TW3
hbr-miR156	hbr-miR156	hbr-miR156
hbr-miR159a	hbr-miR159a	hbr-miR159a
hbr-miR166a	hbr-miR166a	hbr-miR166a
hbr-miR166b	hbr-miR166b	hbr-miR166b
hbr-miR2118	hbr-miR2118	hbr-miR2118
hbr-miR319	hbr-miR319	hbr-miR319
hbr-miR396a	hbr-miR396a	hbr-miR396a
hbr-miR396b	hbr-miR396b	hbr-miR396b
hbr-miR398	hbr-miR398	hbr-miR408a
hbr-miR408a	hbr-miR408a	hbr-miR408b
hbr-miR408b	hbr-miR408b	hbr-miR476
hbr-miR476	hbr-miR476	hbr-miR482a
hbr-miR482a	hbr-miR482a	hbr-miR482b
hbr-miR482b	hbr-miR482b	hbr-miR6168
hbr-miR6168	hbr-miR6168	hbr-miR6169
hbr-miR6169	hbr-miR6170	hbr-miR6172
hbr-miR6172	hbr-miR6171	hbr-miR6173
hbr-miR6173	hbr-miR6172	hbr-miR6174
hbr-miR6174	hbr-miR6173	hbr-miR6484
hbr-miR6484	hbr-miR6174	hbr-miR6485
hbr-miR9386	hbr-miR6484	hbr-miR9386
hbr-miR9387	hbr-miR6485	hbr-miR9387
	hbr-miR9386	

Table S3. Differentially abundant miRNAs from comparisons between TW and NW, OW and NW, and TW and OW.

	miRNA	log2FoldChange	pval	significant*	down*	up*
OWvsNW	novel_101	-3.55329	0.034524	TRUE	√	
	novel_76	-2.24069	0.000252	TRUE	√	
	novel_40	-1.83533	0.031068	TRUE	√	
	novel_166	-1.57919	0.014224	TRUE	√	
	novel_177	-1.35617	0.004354	TRUE	√	
	novel_52	-1.04289	0.010312	TRUE	√	
	novel_28	1.141242	0.024816	TRUE		√
	novel_66	1.498542	0.018609	TRUE		√
	novel_93	3.542923	0.010165	TRUE		√
	novel_86	3.57803	0.001681	TRUE		√
	novel_165	3.88541	0.017341	TRUE		√
TWvsNW	novel_166	-2.41563	0.001977	TRUE	√	
	novel_47	-1.31944	0.021711	TRUE	√	
	novel_28	1.302691	0.024917	TRUE		√
	novel_86	3.098759	0.012327	TRUE		√
	novel_67	3.601821	1.63E-05	TRUE		√
TWvsOW	novel_66	-1.40476	0.036822	TRUE	√	
	novel_67	2.067172	0.039911	TRUE		√

significant*: significant differential expression

down*：down regulation

up*：up regulation

Table S4. Both significant differentially expressed miRNAs and their predicted target genes from comparisons between TW and NW, OW and NW, and TW and OW.

	miRNA	target gene
	novel_166	gene-GH714_039853
	novel_28	gene-GH714_000517
	novel_28	gene-GH714_002615
	novel_28	gene-GH714_004216
	novel_28	gene-GH714_010854
TW vs NW	novel_28	gene-GH714_017700
	novel_28	gene-GH714_020114
	novel_47	gene-GH714_004418
	novel_67	gene-GH714_007987
	novel_67	gene-GH714_027993
	novel_101	gene-GH714_006347
	novel_101	gene-GH714_010180
	novel_101	gene-GH714_014338
	novel_101	gene-GH714_019616
	novel_101	gene-GH714_021337
	novel_101	gene-GH714_023210
	novel_101	gene-GH714_025897
	novel_101	gene-GH714_027813
	novel_101	gene-GH714_028763
	novel_101	gene-GH714_029573
	novel_101	gene-GH714_038685
	novel_101	gene-GH714_039644
	novel_165	gene-GH714_002142
	novel_165	gene-GH714_002173
OW vs NW	novel_165	gene-GH714_019861
	novel_165	gene-GH714_028928
	novel_165	gene-GH714_034456
	novel_166	gene-GH714_039853
	novel_177	gene-GH714_007881
	novel_28	gene-GH714_000517
	novel_28	gene-GH714_002615
	novel_28	gene-GH714_004216
	novel_28	gene-GH714_010854
	novel_28	gene-GH714_017700
	novel_28	gene-GH714_020114
	novel_40	gene-GH714_007097
	novel_66	gene-GH714_016005
	novel_66	gene-GH714_032371
	novel_76	gene-GH714_026108
	novel_76	gene-GH714_035558

	novel_76	gene-GH714_036640
	novel_66	gene-GH714_016005
	novel_66	gene-GH714_024461
	novel_66	gene-GH714_025361
	novel_66	gene-GH714_032371
	novel_66	gene-GH714_033362
TW vs OW	novel_67	gene-GH714_006371
	novel_67	gene-GH714_007987
	novel_67	gene-GH714_009435
	novel_67	gene-GH714_027993
	novel_67	gene-GH714_033677
	novel_67	gene-GH714_042439

Table S5. Pearson's correlation coefficients between miRNAs and their predicted target genes in 300-day reaction wood.

gene1	gene2	pearson−correlation−coefficient	pvalue
novel_28	gene-GH714_000517	-0.852768489	0.003480805
novel_28	gene-GH714_002615	-0.862109416	0.002793852
novel_76	gene-GH714_003160	-0.809546896	0.008194411
novel_28	gene-GH714_004216	-0.846692657	0.003985078
novel_47	gene-GH714_004418	-0.863055401	0.00272998
novel_67	gene-GH714_006371	-0.94412116	0.000128549
novel_40	gene-GH714_007097	-0.847049415	0.003954165
novel_177	gene-GH714_007786	-0.815348116	0.007398135
novel_177	gene-GH714_007881	-0.934378387	0.000223441
novel_67	gene-GH714_007987	-0.841958081	0.004411162
novel_67	gene-GH714_009435	-0.931511953	0.000258768
novel_177	gene-GH714_010038	-0.808471399	0.008348146
novel_28	gene-GH714_010854	-0.865525969	0.002567929
novel_28	gene-GH714_013154	-0.915240123	0.00053686
novel_28	gene-GH714_013930	-0.928707911	0.000296954
novel_101	gene-GH714_014338	-0.814466076	0.00751566
novel_66	gene-GH714_016005	-0.894605922	0.001127283
novel_28	gene-GH714_017700	-0.961979934	3.39953E-05
novel_165	gene-GH714_019861	-0.928372927	0.000301766
novel_28	gene-GH714_020114	-0.944479542	0.000125731
novel_166	gene-GH714_022915	-0.816907696	0.007193398
novel_66	gene-GH714_024461	-0.811299085	0.007948074
novel_66	gene-GH714_025361	-0.922897568	0.000388385
novel_101	gene-GH714_025897	-0.819385427	0.006876099
novel_67	gene-GH714_027993	-0.890357714	0.001288944
novel_66	gene-GH714_032371	-0.875704872	0.001969898
novel_177	gene-GH714_032593	-0.803321191	0.009111459
novel_66	gene-GH714_033362	-0.881070045	0.001697234
novel_67	gene-GH714_033677	-0.80530636	0.008811879
novel_165	gene-GH714_033952	-0.865135029	0.002593117
novel_76	gene-GH714_035558	-0.88011229	0.001743859

novel_76	gene-GH714_036640	-0.856947799	0.003160595
novel_177	gene-GH714_039702	-0.878369531	0.001830962
novel_93	gene-GH714_039737	-0.926545867	0.000328976
novel_166	gene-GH714_039853	-0.82681021	0.005982477
novel_177	gene-GH714_041793	-0.983212967	1.98535E-06
novel_177	gene-GH714_041827	-0.858528184	0.003044989
novel_52	gene-GH714_042278	-0.835329861	0.005058626
novel_67	gene-GH714_042439	-0.844432312	0.004184797
